# Intrapartum ultrasound assessment of cervical dilatation and its value in detecting active labor

**DOI:** 10.1007/s40477-018-0309-2

**Published:** 2018-07-28

**Authors:** Yaw A. Wiafe, Bill Whitehead, Heather Venables, Edward T. Dassah, Torbjørn M. Eggebø

**Affiliations:** 1 College of Health and Social Care, University of Derby, Derby, UK; 2 Department of Sonography, College of Health Sciences, Kwame Nkrumah University of Science and Technology, Kumasi, Ghana; 3 Department of Obstetrics and Gynecology, Komfo Anokye Teaching Hospital and School of Public Health, Kwame Nkrumah University of Science and Technology, Kumasi, Ghana; 4 National Center for Fetal Medicine, Trondheim University Hospital (St Olavs Hospital), Trondheim, Norway; 5 Department of Laboratory Medicine, Children’s and Women’s Health, Norwegian University of Science and Technology, Trondheim, Norway; 6 Department of Obstetrics and Gynecology, Stavanger University Hospital, Stavanger, Norway

**Keywords:** Transperineal ultrasound, Digital vaginal examination, Cervical dilatation, Active labor

## Abstract

**Introduction:**

We aimed to examine the agreement between ultrasound and digital vaginal examination in assessing cervical dilatation in an African population and to assess the value of ultrasound in detecting active labor.

**Method:**

A cross-sectional study was conducted in a teaching hospital in Ghana between April and September of 2016. Anterior–posterior and transverse diameters of cervical dilatation were measured with ultrasound and the mean value was compared with digital vaginal examination in 195 women in labor. Agreement between methods was examined with correlation coefficients and with Bland–Altman plots. Active labor was defined when cervix was dilated ≥ 4 cm with vaginal examinations. ROC curve analysis was conducted on the diagnostic performance of ultrasound in detecting active labor.

**Results:**

Data were analyzed in 175 out of 195 (90%) cases where ultrasound could clearly visualize the cervix. The remaining 20 cases were all determined by digital vaginal examination as advanced cervical dilatation (≥ 8 cm), advanced head station (≥ + 2), and with ruptured membranes. The Pearson correlation coefficient (*r*) was 0.78 (95% CI 0.72–0.83) and the intra-class correlation coefficient was 0.76 (95% CI 0.69–0.81). Bland–Altman analysis obtained a mean difference of − 0.03 cm (95% CI − 0.18 to 0.12) with zero included in the CI intervals, indicating no significant difference between methods. Limits of agreement were from − 2.01 to 1.95 cm. Ultrasound predicted active labor with 0.87 (95% CI 0.75–0.99) as the area under the ROC curve.

**Conclusion:**

Ultrasound measurements showed good agreement with digital vaginal examinations in assessing cervical dilatation during labor and ultrasound may be used to detect active labor.

## SOMMARIO

**Introduzione:**

L’obiettivo del nostro studio è stato quello di esaminare la relazione fra ecografia ed esplorazione vaginale nella valutazione della dilatazione della cervice in una popolazione africana, e di valutare l’utilità degli ultrasuoni nel rilevare il travaglio attivo.

**Metodi:**

È stato condotto uno studio cross-sectional in un ospedale universitario in Ghana tra Aprile e Settembre 2016. Sono stati misurati i diametri anteriore-posteriore e trasversale della dilatazione cervicale con metodica ecografica ed il valore medio è stato confrontato con l’esplorazione vaginale in 195 donne in travaglio. La concordanza fra le metodiche è stata esaminata con i coefficienti di correlazione e con l’analisi di Bland–Altman. Il travaglio è stato definito attivo in presenza di una dilatazione della cervice maggiore di 4 cm alla esplorazione vaginale. L’analisi della curva ROC è stata eseguita per le prestazioni diagnostiche ecografiche nel rilevamento del travaglio attivo.

**Risultati:**

I dati sono stati analizzati in 175/195 (90%) dei casi in cui l’ecografia è stata in grado di visualizzare chiaramente la cervice. I restanti 20 casi sono stati tutti determinati con l’esplorazione vaginale come dilatazione cervicale avanzata (≥ 8 cm), posizione di testa avanzata (≥ +2) e rottura delle membrane. Il coefficiente di correlazione di Pearson (r) era 0,78 (IC 95% 0,72 - 0,83) e il coefficiente di correlazione intra-classe era 0,76 (IC 95% 0,69 - 0,81). L’analisi di Bland–Altman ha ottenuto una differenza media di -0,03 cm (95% CI da -0,18 a 0,12) con lo zero incluso negli intervalli di CI, non indicando differenze significative tra i metodi. I limiti di confidenza erano da -2,01 cm a 1,95 cm. L’ecografia ha predetto il travaglio attivo con 0,87 (IC 95% 0,75-0,99) come area sotto la curva ROC.

**Conclusioni:**

Le misurazioni ecografiche hanno mostrato un buon accordo con la valutazione digitale della dilatazione cervicale durante il travaglio, e possono essere utilizzate per rilevare il travaglio attivo.

## Introduction

Diagnosing the onset of active labor is one of the most important but difficult judgments made by providers of maternity care [1]. Cervical dilatation is the most objective measure for diagnosing active labor, and the fundamental reason for performing digital vaginal examination (VE) in women presenting with signs of labor [2–4]. The limitations of digital VE include increased risk of infection and the fact that it is uncomfortable for some women [5–7].

In recent times, however, a number of studies have demonstrated the effectiveness of using ultrasound in determining cervical dilatation, fetal head position, head station, and engagement [8, 9].

The determination of cervical dilatation with ultrasound was first reported in an Israeli study by Zimmerman et al. [10]. They used 3D acquisitions and found significant correlation between ultrasound and clinical examination in the determination of cervical dilatation. Consequently, they suggested that ultrasound could become a useful adjunct for monitoring selected cases of labor. However, they recommended that since their findings were only preliminary, further research should be encouraged in specific clinical settings by employing different study designs [10]. Prior to this initial study, it was thought that the visualization of cervical dilatation on ultrasound was unlikely to be successful. Specifically, it was felt that the lumen of the dilating cervix might be obscured by the bony part of the descending fetal head [11]. However, Hassan et al. later published a 2D method of visualizing cervical dilatation using a smaller sample population of 21 in their first study [12] and 20 in their follow-up study [13]. Subsequently, Yuce et al. [14] and Benediktsdottir et al. [15] replicated similar studies in their respective Turkish and Swedish study populations. They all obtained significant correlation between ultrasound and digital VE findings on cervical dilatation.

Clearly, since digital VE determination of cervical dilatation plays a major role in the determination of active labor, further research needed to be conducted to assess the value of ultrasound in detecting active labor. Consequently, this study was conducted in an African population to assess the agreement between ultrasound and digital VE on cervical dilatation and to determine the value of ultrasound in detecting active labor.

## Methods

This cross-sectional study was conducted between April and September of 2016 at the labor and delivery ward of the Komfo Anokye Teaching Hospital in Kumasi, Ghana. The study was approved by the Committee for Human Research Publication and Ethics of the Komfo Anokye Teaching Hospital and informed consent was obtained from all study participants. The inclusion criteria were singleton gestation, cephalic presentation, and spontaneous labor. The exclusion criteria were induced labor, breech presentation, multiple pregnancy, polyhydramnios, known fetal abnormality, and previous cesarean section.

Digital VE was performed by the managing clinician on duty, who determined the cervical dilatation upon admission to the labor ward. Only obstetricians with at least 5 years of experience in intrapartum care were allowed to determine the cervical dilatation. In addition to determining cervical dilatation, they established whether the woman was in active labor, based on the facility’s criteria of ≥ 4 cm cervical dilatation whether it was effaced.

Immediately after performing the digital VE, transperineal ultrasound was performed by an independent ultrasound investigator with over 10 years of experience in ultrasound imaging, who was blinded to the digital VE findings. The ultrasound system used was a mobile unit (P 300, Siemens Acuson, Italy) that was equipped with a 2–5 MHz curvilinear transducer. All scans were performed between uterine contractions with a sterile glove on the right hand; the transducer was also covered with a sterile glove, which served as a barrier for preventing cross infection. The transducer was washed with soap under running water after each scan.

**Fig. 1:**
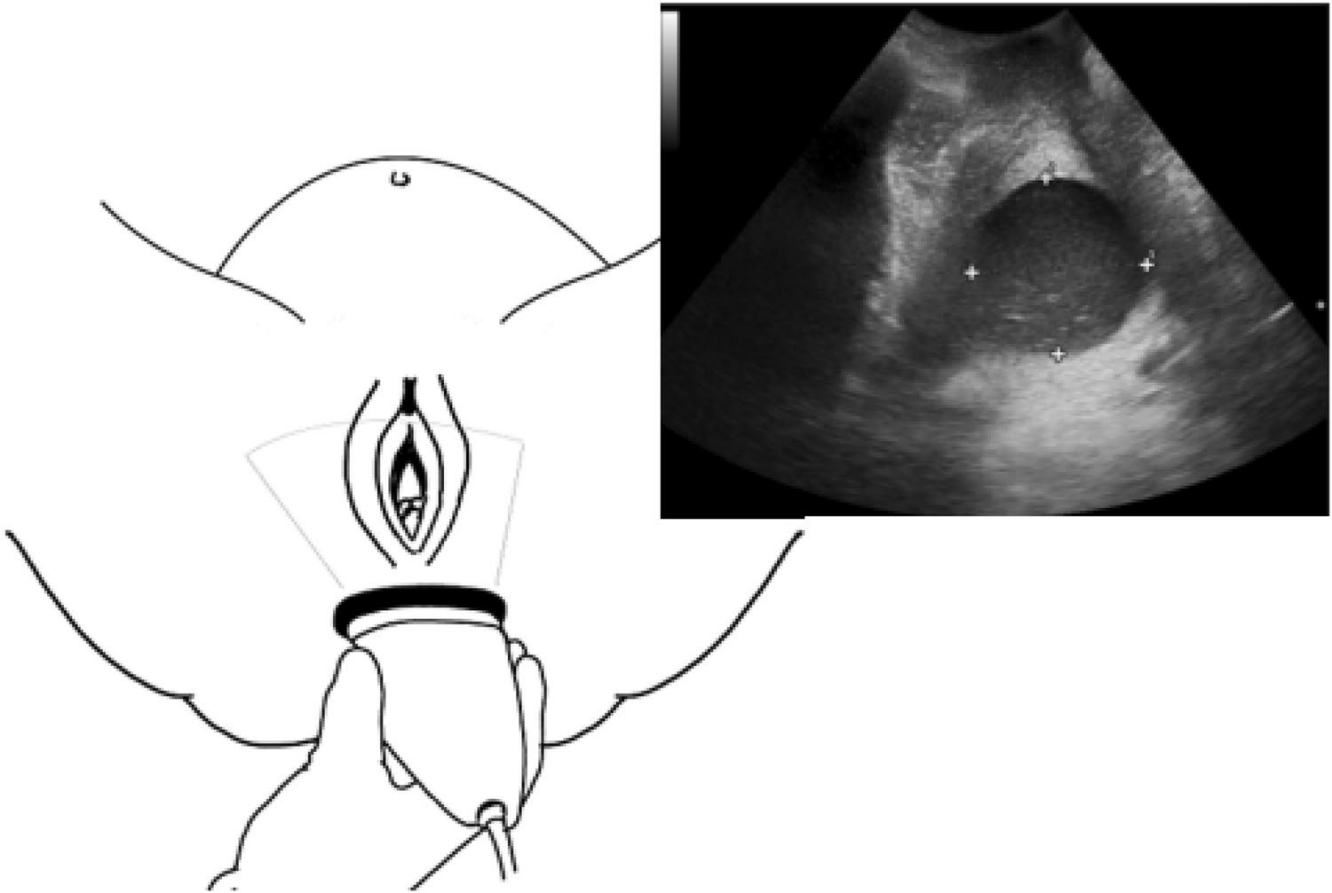
Transverse probe orientation showing ultrasound beam direction in obtaining cervical dilatation

**Fig. 2:**
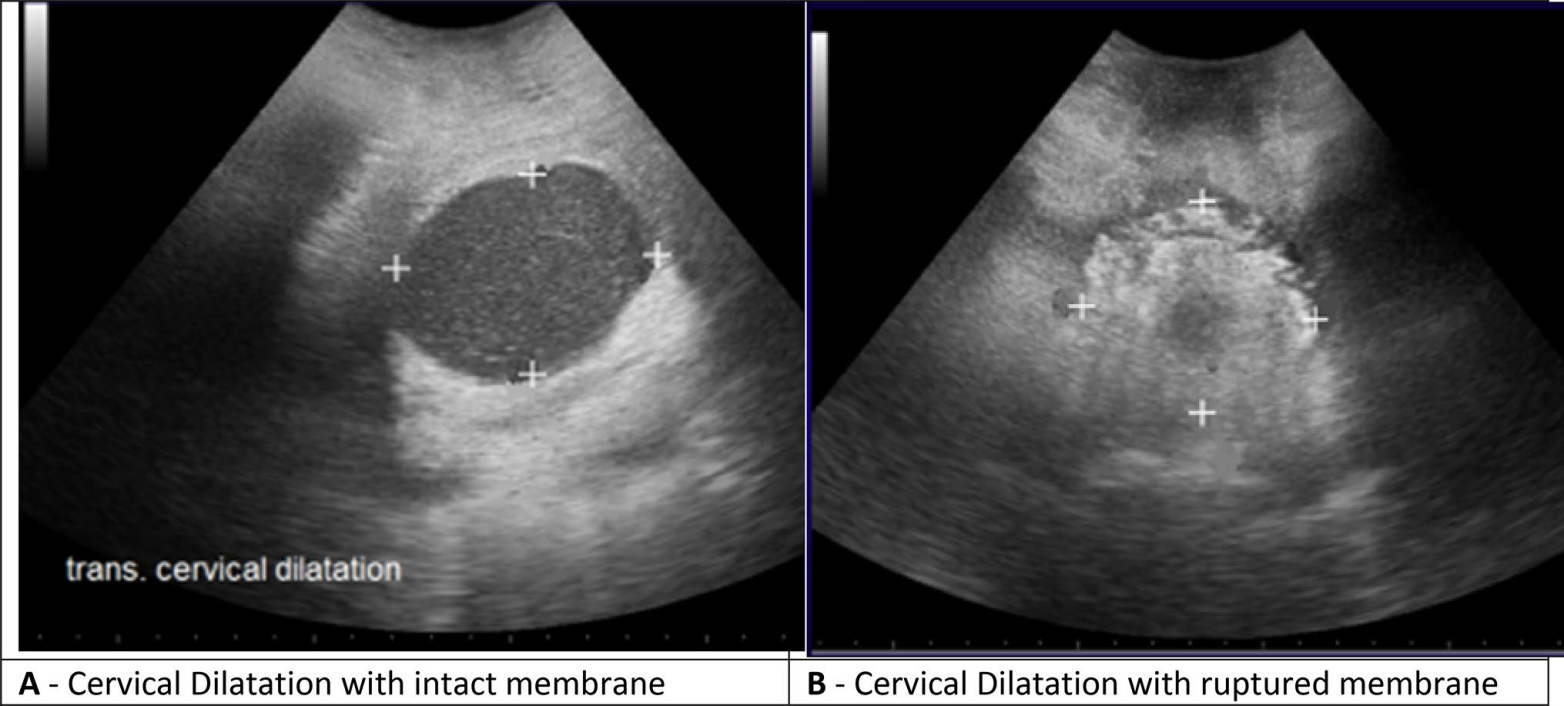
Cervical dilatation with intact and ruptured membranes

The transperineal ultrasound examination was performed by placing the curvilinear transducer at the perineal space between the labia and the anus. With the transducer held in the sagittal plane over the perineal region, the fetal head was displayed on the monitor, often with part of the symphysis pubis showing. The transducer was rotated 90^o^ counterclockwise for a transverse view of the region. Afterwards, the transducer was tilted posteriorly to visualize the rectum and then gradually tilted back anteriorly until the rectum was kept out of view. In tilting the transducer towards the anterior direction, the next clearly visualized region after the rectum was the cervix (Fig. 1). However, an extreme anterior tilt was avoided, as it resulted in missing the dilating cervix or obtaining it from a plane that was far too high. The sonographic appearance of the dilating cervix depended on whether the membranes of the woman were ruptured (Fig. 2). In cases where the membranes were not ruptured, the sonographic appearance also depended on the extent of head descent, which may show some hair on the fetal head within the cervical lumen, or the amniotic fluid content, which may also show internal echoes in the cervical lumen. After visualizing the cervix, measurements were obtained from the anterior–posterior (AP) diameter and the right-to-left (transverse) diameter of the cervix.

### Statistical analyses

Linear regression analysis was performed to investigate the association between ultrasound measurements of the cervix and digital examination. Correlations were expressed using the Pearson correlation coefficient (*r*) and the intra-class correlation coefficient (ICC). Inter-method agreement was examined between digital examinations and ultrasound measured AP diameter, transverse diameter, and mean diameter. To assess systematic bias between ultrasound measurements and digital examination, differences between values were plotted against the means of the measurements. If zero was within the 95% CI of the difference, no bias was assumed. Limits of agreement with 95% CIs of the lower and upper limits were calculated as described by Bland and Altman [16]. Active labor was defined when digital examination found the cervix to be dilated ≥ 4 cm. Ultrasound prediction of active labor was analyzed with ROC curves. The data were analyzed with the statistical software package SPSS Statistics version 25.0 (IBM SPSS, Armonk, NY, USA) and Vassarstats (http://vassarstats.net).

## Results

Out of the total 201 parturients who consented to participate in the study, complete data were obtained from 195 participants who were between the ages of 18 and 39 years, with a mean age of 27 years. This comprised 47% nulliparous, 22% primiparous, and 31% multiparous. Mean gestational age was 39 weeks + 4 days and mean BMI was 28 kg/m^2^ (range 20–42 kg/m^2^).

Ultrasound could clearly visualize the cervix in 175 (90%) of the cases. The remaining 10%, which were difficult to visualize on ultrasound, were all determined by clinical examination as having advanced cervical dilatation (≥ 8 cm), advanced head station (≥ + 2), and ruptured membranes. The statistical analysis of the agreement between ultrasound and clinical examination was therefore based on the 175 cases of cervical dilatation that were clearly visualized on ultrasound.

**Fig. 3:**
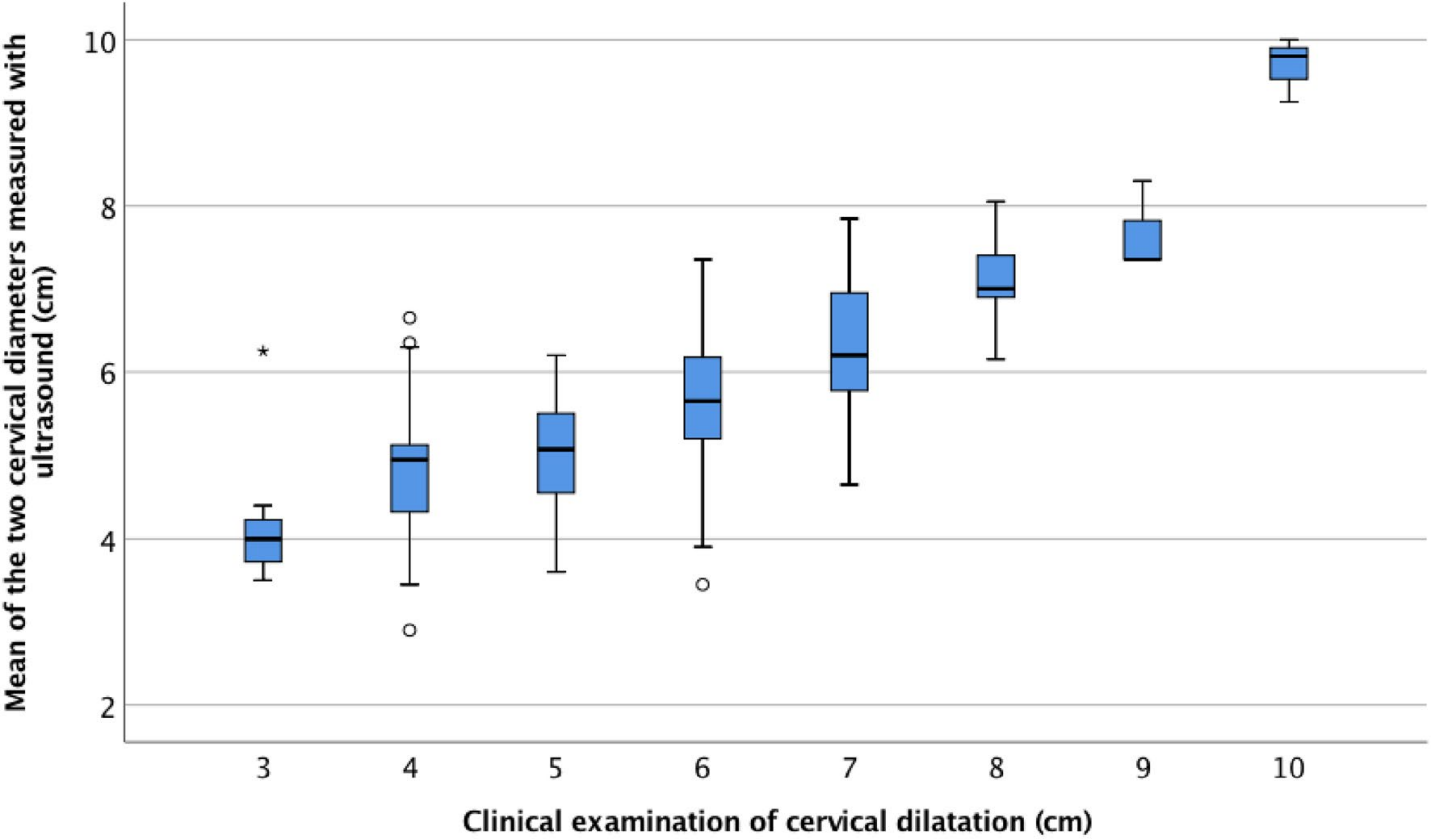
Box plot illustrating the association between digital vaginal examinations and ultrasound measurements

**Fig. 4:**
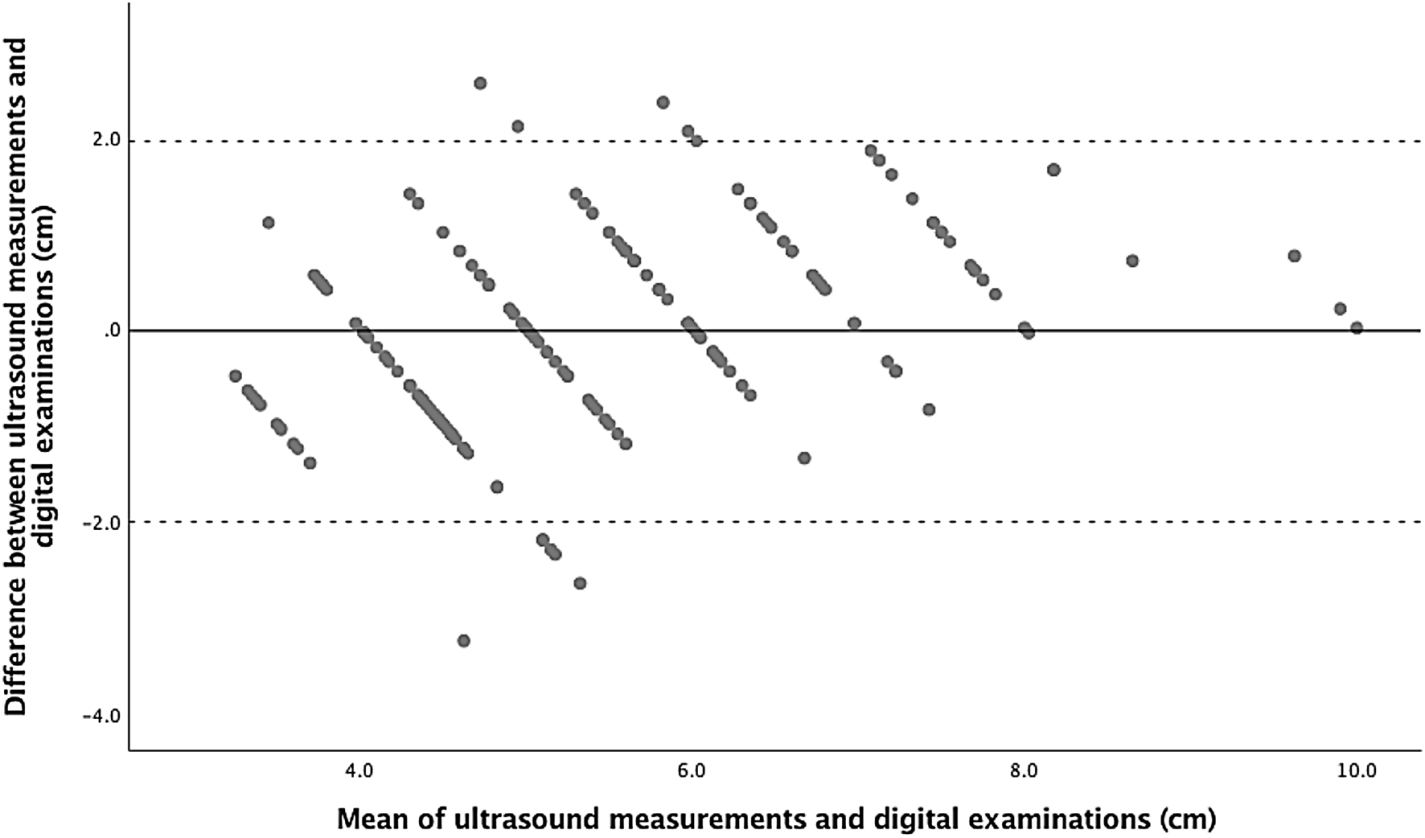
Bland–Altman plot illustrating agreement between digital vaginal examinations and ultrasound measurements of cervical dilatation. Mean difference and limits of agreement are shown

The association between digital VE and the mean of AP and transverse ultrasound diameters is presented as a box plot in Fig. 3, which shows significant correlation. The Pearson correlation coefficient (*r*) was 0.78 (95% CI 0.72–0.83) and the ICC was 0.76 (95% CI 0.69–0.81). The mean difference was -0.03 cm (95% CI − 0.18 to 0.12). Zero was included in the CI intervals, indicating no significant difference between methods. Limits of agreement were from − 2.01 to 1.95 cm. Details of agreement are presented in Table 1 and further illustrated in a Bland–Altman plot (Fig. 4). In addition, Table 1 shows the agreement between the ultrasound AP diameter and clinical examination, and the agreement between the ultrasound transverse diameter and clinical examination.

**Table 1:** Inter-method agreement between ultrasound examinations (anterior–posterior diameter, transverse diameter, and mean diameter) and digital examination

	Cervix dilatation (cm)			Inter-CC (95% CI)	Difference between the two methods (cm)							
	Mean	Median	Range		Mean	95% CI of mean	1.96 SD	Lower limit	Upper limit	95% CI of lower limit	95% CI of upper limit	Range
AP diameter	5.5	5.3	2.7–9.8	0.73 (0.66–0.79)	0.01	− 0.15 to 0.17	2.10	− 2.09	2.11	− 2.36 to − 1.82	1.84–2.38	− 3.2 to 2.6
Transverse diameter	5.6	5.4	3.1–10	0.75 (0.68–0.81)	− 0.07	− 0.22 to 0.08	2.02	− 2.09	1.95	− 2.35 to − 1.69	1.69–2.21	− 3.3 to 2.5
Mean diameter	5.5	5.3	2.9–10	0.76 (0.69–0.81)	− 0.03	− 0.18 to 0.12	1.98	− 2.01	1.95	− 2.27 to − 1.74	1.69–2.21	− 3.3 to 2.6

Mean, median and range for cervical dilatation are calculated from the mean of the two methods

*Inter-CC* intra-class correlation coefficient, *SD* standard deviation, *AP diameter* anterior–posterior diameter

The vaginally examined cervical dilatation was ≥ 4 cm in 164 of the 175 (94%) women. Ultrasound measurements (mean of the two diameters) predicted active labor with 0.87 (95% CI 0.75–0.99) as the area under the ROC curve.

## Discussion

The main finding in our study was a good agreement between ultrasound and digital VE on cervical dilatation. We also found that ultrasound examination of cervical dilatation can be used for diagnosing active labor.

The preliminary research findings of Zimmerman et al. [10] were groundbreaking as ultrasound measurement of cervical dilatation was previously considered to be difficult. However, their use of 3D rather than 2D had disadvantages, as only locations with 3D/4D systems could use ultrasound for detecting cervical dilatation. This excludes the numerous settings around the globe that are using 2D ultrasound systems. 3D/4D acquisitions are also time consuming to analyze and inconvenient to use online in the labor ward. Fortunately, Hassan et al. [12] came across the dilating cervix on 2D, during a study on using ultrasound for assessing fetal head decent.

Our study is the first to be conducted in a Black African population as no similar study was found in this population by a recent systematic review and meta-analysis [8]. It is also the largest study comparing ultrasound measurements and VEs of cervical dilatation. The correlation coefficient obtained by the present study (*r* = 0.78) is highly comparable to the findings of previous studies that were conducted in smaller sample populations. Hassan et al. [12, 13] obtained their measurements in the AP dimension and found the Pearson correlation coefficient to be 0.82. Zimmerman et al. [10] and Benediktsdottir et al. [15] obtained their measurements from the mean of the AP and transverse diameters. The AP diameter is easiest to measure with ultrasound, but we agree with Zimmerman et al. and Benediktsdottir et al. that the mean of two diameters is preferred. The ICC in our study (0.76) was also comparable to the ICC in Benediktsdottir’s (0.83) and Yuce et al.’s (0.82). We did not find any significant difference between ultrasound measurements and clinical examinations, which contrasts results from previous studies where ultrasound measurements were significantly smaller than clinical examination. In previous studies, the authors supposed that this difference was due to stretching of the cervix in clinical examinations. The tradition in how to assess cervical dilatation with VEs may differ between birth attendants and different clinical settings. We think that ultrasound is a more objective method.

The comparability of the current study with previous ones carried out across continents in a variety of study populations and clinical settings and by independent investigators attests to the reliability of ultrasound as an alternative method for estimating cervical dilatation. In addition, the present study demonstrated a high diagnostic performance for the diagnosis of active labor. This is the first time the diagnostic performance of ultrasound in detecting active labor has been reported. This new finding therefore suggests that the diagnosis of active labor could be made with ultrasound in the triage area before labor ward admission. The International Society of Ultrasound in Obstetrics and Gynecology (ISUOG) recommends six basic ultrasound steps, which include examining the number of fetuses, fetal heart rate, fetal presentation, placental location, amniotic fluid assessment, and basic biometry. These examinations are especially needed at admission to the labor ward in low resource countries where some women may not have been examined with ultrasound during their pregnancy. Measurement of cervical dilatation may be added to confirm active labor. This practice may, however, require basic ultrasound training for labor ward personnel, which could be explored by further research.

Historically, transperineal ultrasound successfully became the alternative to clinical examination in assessing the cervical length of third trimester pregnancies, after Mahony et al. [17] reported a high correlation between transperineal ultrasound and clinical examination in assessing cervical length. However, unlike the cervical length, which is measured in the sagittal view, the cervical dilatations of recent studies were all measured from the transverse view. We found the cervical dilatation easier to identify in the transverse view when the technique and anatomical landmarks described above were followed (Fig. 1).

One limitation of ultrasound in this study was that it was unable to visualize the cervix when the cervical dilatation was highly advanced (i.e., ≥ 8 cm) in ruptured membrane cases. It could, however, determine the cervix at advanced cervical dilatations when the membranes were intact. It therefore suggests that ultrasound may not be useful for assessing cervical dilatation in cases of advanced cervical dilatation when the membranes are ruptured. However, given the non-invasive approach of ultrasound in comparison to digital VE, ultrasound could be used as the initial tool for detecting cervical dilatation before digital VE is considered. This could limit the use of digital VE in assessing cervical dilatation to only cases that cannot be visualized by ultrasound, thus minimizing the likelihood of maternal discomfort and the risk of infection that are associated with digital VE [7]. Another limitation is that most women were in active labor when examined. In high resource countries, women are often referred to the labor ward in the latent phase; in contrast, African women often arrive late. This reduces the external validity of our study in defining active labor with ultrasound. We acknowledge that our study design for the detection of active labor by ultrasound is not optimal. The preferred method will be a randomized study in which cervical dilatation is diagnosed with ultrasound in one group and diagnosed with clinical examination in another group, to determine the technique which best identifies patients entering labor. Precision, frequency of chorioamnionitis, and maternal preferences should be examined in a new study.

In conclusion, ultrasound measurements show good agreement with digital VEs in assessing cervical dilatation during labor and that ultrasound may be used to detect active labor.
